# Biomarkers of Exposure among USA Adult Hookah Users: Results from Wave 1 of the Population Assessment of Tobacco and Health (PATH) Study (2013–2014)

**DOI:** 10.3390/ijerph17176403

**Published:** 2020-09-02

**Authors:** Mark J. Travers, Cheryl Rivard, Eva Sharma, Sandra Retzky, Berran Yucesoy, Maciej L. Goniewicz, Cassandra A. Stanton, Jiping Chen, Priscilla Callahan-Lyon, Heather L. Kimmel, Baoyun Xia, Yuesong Wang, Connie S. Sosnoff, Víctor R. De Jesús, Benjamin C. Blount, Stephen S. Hecht, Andrew Hyland

**Affiliations:** 1 Department of Health Behavior, Roswell Park Comprehensive Cancer Center, Buffalo, NY 14203, USA; mark.travers@roswellpark.org (M.J.T.); Maciej.Goniewicz@RoswellPark.org (M.L.G.); Andrew.Hyland@RoswellPark.org (A.H.); 2 Westat, Rockville, MD 20850, USA; EvaSharma@westat.com (E.S.); cassandrastanton@westat.com (C.A.S.); 3 Food and Drug Administration, Center for Tobacco Products, Calverton, MD 20705, USA; Sandra.Retzky@fda.hhs.gov (S.R.); Berran.Yucesoy@fda.hhs.gov (B.Y.); Jiping.Chen@fda.hhs.gov (J.C.); priscilla.callahan-lyon@fda.hhs.gov (P.C.-L.); 4 National Institute on Drug Abuse, National Institutes of Health, Bethesda, MD 20892, USA; heather.kimmel@nih.gov; 5 Division of Laboratory Sciences, National Center for Environmental Health, Centers for Disease Control and Prevention, Atlanta, GA 30333, USA; Vvq2@cdc.gov (B.X.); uxi5@cdc.gov (Y.W.); css3@cdc.gov (C.S.S.); FOA5@cdc.gov (V.R.D.J.); bkb3@cdc.gov (B.C.B.); 6 Masonic Cancer Center, University of Minnesota, Minneapolis, MN 55455, USA; hecht002@umn.edu

**Keywords:** hookah 1, biomarkers 2, tobacco 3

## Abstract

Hookah smoking has become common in the USA, especially among young adults. This study measured biomarkers of exposure to known tobacco product toxicants in a population-based sample of exclusive, established hookah users. Urinary biomarker data from 1753 adults in Wave 1 of the Population Assessment of Tobacco and Health (PATH) Study were used to compare geometric mean concentrations of biomarkers of exposure in exclusive, established past 30-day hookah users to never users of tobacco. Geometric mean ratios were calculated comparing hookah user groups with never users adjusting for age, sex, race/ethnicity, education, past 30-day marijuana use, secondhand smoke exposure and creatinine. Past 30-day hookah users (*n* = 98) had 10.6 times the urinary cotinine level of never tobacco users. Compared to never tobacco users, past 30-day hookah users had 2.3 times the level of the carcinogen 4-(methylnitrosamino)-1-(3-pyridyl)-1-butanol (NNAL), a metabolite of the tobacco-specific nitrosamine (TSNA) 4-(methylnitrosamino)-1-(3-pyridyl)-1-butanone (NNK), 1.3 times higher polycyclic aromatic hydrocarbons (PAHs) 3-hydroxyfluorene and 1-hydroxypyrene, 1.8 times higher levels of acrylonitrile, 1.3 times higher levels of acrylamide, and 1.2 times higher levels of acrolein exposure. These data indicate that hookah use is a significant source of exposure to nicotine, carcinogens, and respiratory toxicants.

## 1. Introduction

Hookah, also known as waterpipe, is a form of tobacco use that is increasingly common in the USA [1]. Wave 1 of the Population Assessment of Tobacco and Health (PATH) Study shows that current use of hookah at the population level in USA adults in 2013–2014 was 4.2% overall and was significantly higher among young adults (18–24 years) at 18.2% [2]. Other, smaller studies have found high prevalence of hookah use among USA college students, with past-year use ranging from 22% to 40% [3].

In different cultures and countries across the globe, there is different terminology for hookah smoking. A narghile or hookah is used to smoke a specially made tobacco mixture also known as shisha or maassel, containing shredded tobacco, propylene glycol and glycerin, sweeteners, and flavorings, typically by indirectly heating the maassel with lighted charcoal. There are tobacco-free maassel mixtures that may be used by hookah cafes to circumvent indoor smoking restrictions on tobacco products [1,4,5,6]. Hookah tobacco smoke (HTS) is a mixture of charcoal combustion products and aerosol from heated maassel that passes through water or other liquids and then through a hose to the user.

Current research on the health effects of hookah smoking is limited, as it is mostly cross-sectional or retrospective, has poor assessment of frequency and duration of use, limited assessment of demographics and dual and poly tobacco use as confounders, and often fails to report the specific type of hookah tobacco used. However, several reviews of the literature on hookah smoking and health effects have been performed. Hookah smoking has been associated with carbon monoxide intoxication [7], cancers (lung [8,9], esophageal [8,9], and bladder [8]), pulmonary (chronic bronchitis, emphysema [7]) and cardiovascular diseases (coronary artery disease [8,10], increased heart rate and high blood pressure [7]). Several studies have also reported a link between hookah smoking and periodontal disease, obstetrical complications, osteoporosis, and mental health problems [7].

Biomarkers of tobacco exposure characterize actual human exposure to harmful or potentially harmful chemicals (HPHCs) resulting from tobacco use [11]. Analytical studies have demonstrated that HTS contains significant amounts of many of the same toxicants found in cigarette smoke such as carbon monoxide (CO), nicotine, tar (nicotine-free particulate matter), polycyclic aromatic hydrocarbons (PAHs), tobacco-specific nitrosamines (TSNAs), and heavy metals [12] such as cobalt, chromium, nickel, cadmium, and lead. Some of these toxicants are known carcinogens (cadmium, lead, the PAH naphthalene), respiratory toxicants (cadmium and lead), and reproductive or developmental toxicants (nicotine, cadmium, and lead) [13]. Monzer et al. demonstrated that most PAHs in HTS are derived from the charcoal used as a heat source [14]. Several clinical studies have corroborated these findings in HTS, demonstrating hookah smoking results in greater exposure to CO, high molecular weight PAHs such as phenanthrene and pyrene and the volatile organic compound (VOC) benzene; similar exposure to nicotine; and lower but significant exposure to TSNAs [15,16] compared to cigarette smoking. Most studies on hookah smoking report findings based on small selected samples of adults. In general, the scientific literature on exposure biomarkers and hookah smoking is limited.

To expand the knowledge about exposure from hookah smoking, this study used data from PATH Study Wave 1. This is the largest study to date assessing exposure to nicotine and other toxicants among exclusive, established hookah users. The primary aim of the study was to estimate urinary concentrations of biomarkers of exposure stratified by two groups: exclusive, established past 30-day hookah users, and never users of tobacco.

## 2. Materials and Methods

### 2.1. Study Population

Participants were from the PATH Study, a nationally-representative, longitudinal cohort study of tobacco use and health in the United States. The National Institutes of Health, through the National Institute on Drug Abuse, is partnering with the U.S. Food and Drug Administration’s Center for Tobacco Products to conduct the PATH Study under a contract with Westat. Data are from the Wave 1 Restricted Use Files (RUF) and from the Wave 1 Biomarker Restricted Use Files (BRUF). The first wave of the PATH Study included 45,971 people aged 12 years and older including 32,320 adults age 18 and older. A stratified probability sample of 11,522 adults who completed the Wave 1 Adult Interview and provided a urine specimen were selected for laboratory analyses. The sample was selected to ensure respondents represented diverse tobacco product use patterns, including users of multiple tobacco products and never users of any tobacco product.

This study was conducted using a subset of 1753 adult participants who completed Wave 1 interviews, provided urine samples for analyses at Wave 1, and met the criteria for our hookah user groups as defined below. Missing data on age, gender, race, Hispanic ethnicity, and adult education were imputed as described in the PATH Study Restricted Use Files User Guide https://doi.org/10.3886/Series606 [17].

### 2.2. Interview Data

The PATH Study used audio-computer assisted self-interviews (ACASI) available in English and Spanish to collect information on tobacco-use patterns and associated health behaviors. Recruitment employed address-based, area-probability sampling, using an in-person household screener to select youths and adults. Adult tobacco users, young adults aged 18 to 24, and African Americans were oversampled relative to population proportions. The weighting procedures adjusted for oversampling and nonresponse; combined with the use of a probability sample, they allow PATH Study estimates to be representative of the non-institutionalized, civilian USA population at the time of Wave 1. The weighted response rate for household screeners was 54.0%. Among households that were screened, the overall weighted response rate was 74.0% for the Adult Questionnaire. Further details regarding the PATH Study design and methods are published elsewhere [18]. Details on survey questionnaire procedures, questionnaires, sampling, weighting, and information on accessing the data are available at https://doi.org/10.3886/Series606 [17]. Westat’s Institutional Review Board approved the study design and data collection protocol.

### 2.3. Demographics and Potential Confounders

The demographic characteristics of the study population that were measured and included in this analysis are sex (male, female), age group (18–21, 22–24, and 25 and over), race/ethnicity (White, non-Hispanic; Black, non-Hispanic; Asian, non-Hispanic; Other race, non-Hispanic; Hispanic), and educational attainment (less than high school or General Education Diploma (GED), high school diploma, and some college or higher). Since secondhand tobacco smoke exposure and marijuana use are other possible sources of significant exposure to combustion products, these were also considered as potential confounders. Marijuana use was measured as any reported marijuana use over the past 30 days (yes or no). The number of hours of secondhand smoke exposure in the past 7 days was determined from the following question: “During the past seven days, about how many hours were you around others who were smoking (whether or not you were smoking yourself)? Include time in your home, in a car, at work, or outdoors”.

### 2.4. Tobacco Use Patterns

The tobacco product use patterns that were assessed and included in this analysis are:

Exclusive, established past 30-day hookah users: exclusive, established users (have ever smoked a hookah, have smoked hookah regularly, and smoke hookah every day or some days) who have smoked hookah at least once in the past 30 days and have not smoked or used any other tobacco product in the past 30 days and answered that they did not use nicotine replacement therapy (NRT) “today, yesterday, or the day before yesterday.” There are 98 participants that fit this definition and provided urine samples.

Exclusive, established, recent hookah users: exclusive, established, recent users are a subset of the previous group of exclusive, established past 30-day hookah users who reported smoking hookah in the past 3 days at the time of bio-specimen collection. There are 24 participants that fit this definition and provided urine samples, and the analysis of this sample is exploratory.

Never users of tobacco products: respondents who have never smoked or used any tobacco product and answered that they did not use any NRT “today, yesterday, or the day before yesterday.” There are 1655 participants that fit this definition and provided urine samples.

In addition, we explored the relation between frequency of use variables and biomarkers of tobacco exposure in the exclusive, established past 30-day hookah user group. In the Wave 1 PATH Study questionnaire, participants were questioned on frequency of hookah use in a series of questions: “Which of the following choices best describes your hookah smoking? Usually I smoke hookah…every day, weekly, monthly, every couple of months, or about once a year?” and “On average, about how many times do you smoke hookah in a (month), (week), (day)?”. However, since these questions ask about “On average” hookah use, their responses do not necessarily coincide with the 30 days prior to biological sample collection. We report every day versus some day use as well as the average number of times hookah was smoked in a month, which was divided into 3 categories: 1–2 times per month, 3–10 times per month, and 11 or more times per month. The type of shisha used was also explored with the following series of questions: “What brand of shisha or hookah tobacco (do I did) you (usually I last) smoke?” and, “does this brand of shisha contain tobacco?”

### 2.5. Biospecimen Collection

At Wave 1, full-void urine specimens were self-collected by 21,801 (67.5%) consenting adult participants. For more information on the aliquots created from the urine biospecimens see the PATH Study W1 Biospecimen Urine Collection Procedures [17].

### 2.6. Biomarker Data

Urine specimens were shipped overnight on dry ice to the CDC laboratories where they were stored at −80 °C until ready for laboratory analysis. Biomarkers were measured using highly selected mass spectrometric methods and met the rigorous accuracy and precision requirements of the quality control/quality assurance program of the CDC’s National Center for Environmental Health, Division of Laboratory Sciences [19,20,21,22,23,24,25,26,27,28,29].

### 2.7. Laboratory Methods for Urinary Biomarkers

This study examined 52 biomarkers of exposure to hookah smoking from the following classes of compounds: nicotine metabolites, TSNAs, metals, PAHs, and VOCs. The National Addiction & HIV Data Archive Program (NAHDAP) website for the Biomarker Restricted-Use Files (BRUF) provides lab panel documentation that includes LLOD for each analyte in each panel (https://www.icpsr.umich.edu/icpsrweb/NAHDAP/studies/36840/datadocumentation#) [30]. Table 1 shows the biomarkers of exposure with abbreviations, half-lives, and analytical methods.

Creatinine-corrected values were calculated for urinary biomarkers of respondents with levels of creatinine between 10–370 mg/dL, to avoid the confounding effects of overly diluted urines, or hyper-concentrated urines as a result of altered renal clearance rates [32]. The creatinine-corrected values were calculated as follows: creatinine result was converted into g/mL, and biomarker result was also converted into units per mL; then the biomarker mass was divided by creatinine mass to produce biomarker mass per gram of creatinine.

### 2.8. Statistical Analysis

We first estimated the weighted prevalence of selected demographic characteristics. Data for each biomarker was right skewed, all were transformed using the natural log. Creatinine-corrected geometric means were calculated for each biomarker and are presented as descriptive findings for each tobacco use category; confidence intervals were calculated using the delta method [33]. A generalized linear model was then constructed to compare log-transformed biomarkers of exposure among two user groups, exclusive, established past 30-day hookah users and never tobacco users, adjusting for age, sex, race/ethnicity, education attainment, past 30-day marijuana use and level of second hand smoke (SHS) exposure, with never users of tobacco as the reference category.

As an exploratory analysis, a separate model was created to compare exclusive, established recent hookah users (past three days, a subset of exclusive, established past 30-day users) to never tobacco users; however, given the small sample size of this group, the results are not reported. All multivariable analyses were adjusted for urinary creatinine and used uncorrected biomarker concentrations. Estimates were reported as geometric mean ratios and corresponding 95% confidence intervals of hookah users compared to never tobacco users. For concentrations below the LOD, a value equal to the LOD divided by the square root of two was used in the analysis.

Given that not all respondents agreed to provide biospecimens, the resulting biospecimen assay data represent a subsample of adults, therefore specific urine and blood weights are needed to account for potential differences between the full set of adult interview respondents in the specified tobacco product user groups and the set of adults with analyzed biospecimens. Weighted estimates are representative of never, current, and recent former (within 12 months) users of tobacco products in the USA civilian, noninstitutionalized adult population at the time of Wave 1. These weighting procedures are outlined in the Biospecimen Restricted Use Files User Guide [17]. Analyses were completed using survey (svy) commands in Stata v. 14.0. *p*-values < 0.05 were considered statistically significant.

## 3. Results

### 3.1. Characteristics of the Study Population by Tobacco Use Status

The weighted characteristics of the study population with available biomarker data are shown in Table 2. Compared to never tobacco users, exclusive, established past 30-day hookah users were more likely to be male (57.6% vs. 37.5%, *p* = 0.001), younger (42.5% vs. 10.3% were 18 to 21 years old, *p* < 0.001), more educated (75.6% vs. 58.4% with at least some college, *p* = 0.03), more likely to use marijuana (26.3% vs. 0.6% past 30 day use, *p* < 0.001), and have greater SHS (4.8 vs. 1.9 h in past 7 days, *p* = 0.03). Most hookah users (93.1%) were some day users as opposed to everyday users (6.9%). Mean creatinine levels were significantly lower in never tobacco users compared to hookah users (128.7 vs. 159.1 mg/dL, *p* = 0.002), likely because never tobacco users were older and more likely to be female [34]. Therefore, biomarkers of exposure in Table 3 are presented on a per gram of creatinine basis and subsequent regression models (Table 4) include creatinine as a covariate.

### 3.2. Biomarkers of Exposure by Hookah Use Status

Hookah users had significantly higher concentrations of urinary cotinine and total nicotine equivalents, calculated as the molar sum of cotinine and trans-3′-hydroxycotinine (TNE-2), compared to never tobacco users. Similarly, hookah users had higher concentrations of TSNAs, as measured by NNAL, compared to never tobacco users. Concentrations of lead and cadmium were lower in hookah users compared to never tobacco users. Biomarkers of PAH exposure, 3-hydroxyflourene (3-FLU), and 1-hydroxypyrene (1-PYR) did not differ by tobacco use status. For VOC biomarkers, CYMA (acrylonitrile) concentrations were higher in past 30-day hookah users compared to never tobacco users while HPMA (acrolein) concentrations were not significantly different between tobacco user groups. Unadjusted (except for creatinine) biomarker comparisons between tobacco user groups for select markers are shown in Table 3. Results for the complete set of measured urinary biomarkers can be found in Table S1.

After adjustment for creatinine level (g/mL), age, sex, race/ethnicity, education attainment, past 30-day marijuana use, and level of SHS exposure, past 30-day hookah users had 10.6 times the urinary cotinine level of never tobacco users (Table 4). For TSNAs, past 30-day hookah users had 2.29 times higher NNAL compared to never tobacco users. For PAHs, exclusive, established past 30-day hookah users had 1.28 times higher 3-FLU and 1.33 times higher 1-PYR compared to never tobacco users. For VOCs, exclusive, established past 30-day hookah users had 1.81 times higher levels of the acrylonitrile metabolite CYMA compared to never tobacco users. In these adjusted models, we see no differences in levels of metals cadmium and lead or in the VOC HPMA, between hookah users and never tobacco users. Results with confidence intervals for all measured biomarkers can be found in Table S2.

### 3.3. Relation between Frequency of Product Use and Tobacco-Specific Biomarker Concentrations

**Figure 1:**
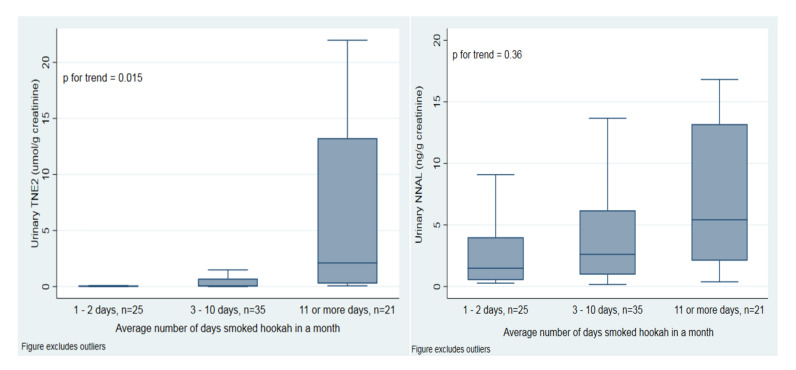
Weighted urinary TNE-2 and NNAL concentrations by average number of times smoked hookah in a month among past 30-day exclusive, established hookah users. Horizontal lines within boxes represent median values. The bottom and top of the boxes are the 25th and 75th percentiles. The distance between the top and bottom boxes represents the interquartile range (IQR). Top and bottom horizontal bars are the maximum and the minimum without outliers. Outliers have been excluded from the figures but remain in the analysis.

Figure 1 shows tobacco-specific biomarkers in the exclusive, established past 30-day hookah user group by average number of times smoked hookah in a month. TNE-2 levels increased with more average times smoked per month (*p* trend = 0.018). NNAL concentrations also appear to be increasing with increasing average number of times smoked per month but this trend is not significant (*p* trend = 0.354).

### 3.4. Exposure Biomarkers by Type of Hookah Tobacco Used

Of the 98 participants that smoked hookah in the past 30 days, 58 (58%) reported their usual or last smoked brand of shisha contains tobacco, 11 (11%) reported it does not contain tobacco, and 29 (29%) reported they did not know or did not report a brand last or usually smoked. Urinary cotinine concentrations in these groups were not significantly different at 7.07, 6.50, and 3.71 µg/g creatinine, respectively. Similarly, there was no significant difference in NNAL concentrations in these groups at 2.09, 3.00, and 2.48 ng/g creatinine, respectively. These results should be interpreted with caution due to low reliability; the coefficient of variation is greater than 30%.

## 4. Discussion

This study provided a unique opportunity to exam34ine hookah use and the relationship to a wide range of biomarkers of exposure in a nationally representative sample of USA adults. Significant differences were found in the characteristics of exclusive hookah users in the USA compared to the general population of never tobacco users in terms of demographics, being younger, more educated, and more likely to be male.

Our study showed that urinary cotinine concentrations were higher in exclusive, established past 30-day hookah users compared to never tobacco users. In addition, urinary NNAL, a carcinogenic metabolite of NNK, was found to be elevated in exclusive, established past 30-day hookah users compared to never tobacco users. After adjustment for confounders, PAH levels (3-FLU and 1-PYR) were found to be elevated in hookah users compared to never tobacco users. Although we have no information on the heat source used, this latter finding is consistent with the report by Monzer et al. [14] that charcoal used as a hookah heat source emits significant amounts of PAH. Similarly, the acrylonitrile exposure biomarker CYMA was elevated in hookah users compared to never tobacco users. This finding is consistent with elevated acrylonitrile exposure resulting from smoking: tobacco smoke contains microgram quantities of acrylonitrile [35] and cigarette smoking is associated with markedly increased urinary CYMA [36]. Biomarkers for acrylamide (AAMA and GAMA) and acrolein (CEMA) were similarly elevated in hookah users compared to never tobacco users. This is consistent with previous studies demonstrating increased exposure to acrolein from hookah smoke [37,38].

No consistent differences in concentrations of metals were observed between hookah users and never tobacco users. Univariate analysis showed lower concentrations of cadmium in hookah users compared to never tobacco users, but this was likely confounded by the large difference in age between these two groups [39]. In fact, after adjusting for covariates, including age, there was no difference in cadmium levels between hookah users and never tobacco users (Table 4).

Among the strengths of this study are its population-based, representative sample of USA hookah users and the breadth and diversity of the large number and types of biomarkers included in the analyses. In addition, given the size of the study population, it was possible to restrict this analysis to those smoking only hookah, thereby reducing potential interference from other tobacco product use.

The main limitation of this study is low frequency and inconsistent hookah use patterns. When a tobacco product is used consistently on a daily basis, such as an established 10 stick per day cigarette smoker, it is reasonable for biomarkers of exposure to be relatively stable regardless of the timing of sampling. Exclusive, established hookah use among adults participating in Wave 1 of the PATH Study was relatively rare, and frequency of use in this study was low. Ninety three percent of past 30-day hookah users were non-daily users, smoking on average 7.6 times per month. Ideally, we would include multiple comparison groups, particularly cigarette smokers in our analysis. However, because of the low frequency of daily hookah users, we did not have a large enough sample to make this comparison, and we were only able to compare hookah users to never tobacco users. Therefore, some estimates should be interpreted with caution, particularly analyses subject to statistical stability concerns. In addition, most past 30-day hookah users (*n* = 1720) used other tobacco products such as cigarettes, e-cigarettes, and cigars. We limited our analysis to exclusive established past 30-day hookah users to reduce the potential interference from other tobacco product use.

Most biomarkers have short half-lives on the order of hours to days. As such, we would expect the timing of biospecimen collection in relation to last hookah use to influence the biomarker levels, and in fact, in exploratory analyses, we saw generally higher levels of biomarkers among recent hookah users compared to past 30-day hookah users. Examination of daily hookah users shows that some biomarkers of exposure appear much higher in this group compared to the more common non-daily hookah user group, but this exploratory analysis was limited by the small sample size (*n* = 7) in the daily user group.

Additional patterns of use, including type and brand of hookah tobacco used and location where hookah is usually smoked, were not evaluated due to small sample size of exclusive hookah users and difficulty in matching the temporality of participant responses to biospecimen sample collection. A significant portion of hookah smoke exposure is expected to come from the charcoal used as a heat source. It is likely that most or all hookah smoking was done with charcoal [14]; however, it was not possible to examine the impact of heat source on measured biomarkers as no information was available on heat source type (e.g., charcoal vs. electric heating) or charcoal type used (e.g., quicklight or traditional). Quicklight charcoal produces higher CO levels because it burns at lower temperatures compared to traditional charcoal [40].

## 5. Conclusions

This is the largest study examining biomarkers of hookah smoking in a nationally representative cohort. These data demonstrate that hookah use is a significant source of exposure to nicotine, TSNAs, PAHs, and VOCs, known carcinogens and/or respiratory toxicants. These findings help understand potential health risks from hookah smoking and provide data to inform regulatory strategies.

## Figures and Tables

**Table 1:** Biomarkers of exposure with abbreviations, half-lives, and analytical methods.

Biomarker of Exposure (Abbreviation)	Half-Life	Analytical Assay Method
**Urinary Nicotine Metabolites ^**		
Cotinine (COTT)	16–18 h	All nicotine metabolites were assessed using two separate isotope dilution high performance liquid chromatography/tandem mass spectrometric (HPLC-MS/MS) methods [20,21]
Nicotine (NICT)	1–2 h	
Cotinine N-oxide (COXT)	N/A	
Nicotine 1′-oxide (NOXT)	N/A	
Norcotinine (NCCT)	N/A	
Nornicotine (NNCT)	N/A	
trans-3′-Hydroxycotinine (HCTT)	6.4 h	
**Minor Tobacco Alkaloids**		
Anabasine (ANBT)	16 h	Same as above
Anatabine (ANTT)	10 h	
**Arsenic and Arsenic Compounds**		
Arsenous Acid	10 h	All arsenic compounds were assessed using high performance liquid chromatography/inductively coupled plasma dynamic reaction cell mass spectrometry (HPLC-ICP-DRC-MS)
Arsenic Acid	10 h	
Dimethylarsinic acid	10 h	
Monomethylarsonic acid	10 h	
**Tobacco Specific Nitrosamines (TSNAs)**		
4-methylnitrosamino)-4-(3-pyridyl)-1-butanol (NNAL)	10.3 days [31]	All TSNAs were assessed using isotope dilution high performance liquid chromatography/atmospheric pressure chemical ionization tandem mass spectrometry (HPLC-MS/MS) [22]
N’-nitrosonornicotine (NNN)	N/A	
N’-nitrosoanatabine (NAT)	N/A	
N’-nitrosoanabasine (NAB)	N/A	
**Metals**		
Beryllium (UBE)	Several years	All metals were assessed using inductively coupled plasma mass spectrometry (ICP-MS) [23,24]
Cadmium (UCD)	13.6 years	
Cobalt (UCO)	Several days	
Manganese (UMN)	39 days	
Lead (UPB)	1–2 months in blood & soft tissues, years to decades in bone	
Strontium (USR)	47.3 h	
Thallium (UTL)	1–3 days	
Uranium (UUR)	24 h	
**Polycyclic Aromatic Hydrocarbons (PAHs)**		
1-Naphthol or 1-hydroxynaphthalene (1-NAP)	4.3 h	All PAHs were assessed using enzymatic hydrolysis, on-line solid phase extraction, and isotope dilution liquid chromatography tandem mass spectrometry [27]
2-Naphthol or 2-hydroxynaphthalene (2-NAP)	9.4 h	
3-Hydroxyfluorene (3-FLU)	8.2 h	
2-Hydroxyfluorene (2-FLU)	2.1 h	
1-Hydroxyphenanthrene (1-PHE)	5.1 h	
1-Hydroxypyrene (1-PYR)	6.0 h	
2-Hydroxyphenanthrene and 3-Hydroxyphenanthrene (2-3PHE)	4.1 h	
**Volatile Organic Compounds (VOCs)**		
2-Methylhippuric acid (2MHA) (Xylene)	34 h	All VOCs were assessed using isotope dilution UPLC-MS/MS [28,29]
3,4-Methylhippuric acid (34MH) (Xylene)	34 h	
N-Acetyl-S-(2-carbamoylethyl)-L-cysteine (AAMA) (Acrylamide)	11 or 17.4 h	
N-Acetyl-S-(N-methylcarbamoyl)-L-cysteine (AMCA) (N,N-Dimethylformamide/isocyanates)	23 h	
N-Acetyl-S-(benzyl)-L-cysteine (BMA) (Toluene)	<10 h	
N-Acetyl-S-(2-carboxyethyl)-L-cysteine (CEMA) (Acrolein)	N/A	
N-Acetyl-S-(1-cyano-2-hydroxyethyl)-L-cysteine (CYHA) (Acrylonitrile)	N/A	
N-Acetyl-S-(2-cyanoethyl)-L-cysteine (CYMA) (Acrylonitrile)	8 h	
N-Acetyl-S-(3,4-dihydroxybutyl)-L-cysteine (DHBM) (1,3 Butadiene)	N/A	
N-Acetyl-S-(2-carbamoyl-2-hydroxyethyl)-L-cysteine (GAMA) (Acrylamide)	19 or 25.1 h	
N-Acetyl-S-(2-hydroxyethyl)-L-cysteine (HEMA) (Acrylonitrile, vinyl chloride, ethylene oxide)	>5 h	
N-Acetyl-S-(2-hydroxypropyl)-L-cysteine (HPM2) (Propylene Oxide)	N/A	
N-Acetyl-S-(3-hydroxypropyl)-L-cysteine (HPMA) (Acrolein)	N/A	
N-Acetyl-S-(3-hydroxypropyl-1-methyl)-L-cysteine (HPMM) (Crotonaldehyde)	N/A	
N-Acetyl-S-(4-hydroxy-2-methyl-2-buten-1-yl)-L-cysteine (IPM3) (Isoprene)	N/A	
Mandelic acid (MADA)	2.1, 3.6, or 3.9 h	
N-Acetyl-S-(4-hydroxy-2-buten-1-yl)-L-cysteine (MHB3) (1,3 Butadiene)	>9 or <6 h *	
Phenylglyoxylic acid (PGHA) (Ethylbenzene, styrene)	8.1, 8.8 or 10.5 h	
N-Acetyl-S-(phenyl)-L-cysteine (PMA) (Benzene)	9.1 h	
2-Thioxothiazolidine-4-carboxylic acid (TTCA) (Carbon Disulfide)	8 h	

* Estimated from animal study. Multiple values indicate areas in which the literature lists multiple half-life values. N/A = “Not Available”. ^ Assays were conducted for each of the seven major nicotine metabolites listed. In addition to the nicotine metabolites listed above, “Total Nicotine Equivalents” (TNE-2) was computed and included in statistical analyses. TNE-2 was calculated as the molar sum of cotinine and trans-3′-Hydroxycotinine. For arsenic and arsenic compounds, individual assays were conducted for each of the four listed compounds. The analysis used a summary variable, “Total Inorganic Arsenic”, representing the sum of the arsenous acid, arsenic acid, dimethylarsinic acid, and monomethylarsonic acid levels in each urine sample. As these are summary variables, TNE-2 and Total Inorganic Arsenic do not have listings for limits of detection.

**Table 2:** Weighted sample characteristics * (*n* = 1753).

Characteristics	Never Users of Tobacco Products (*n* = 1655)				Exclusive, Established, Past 30-Day Hookah User (*n* = 98)			
	*N*	%	95% Confidence Interval		*N*	%	95% Confidence Interval	
			**Lower**	**Upper**			**Lower**	**Upper**
**Sex**								
Male	607	37.5	34.9	40.1	53	57.6	46.6	67.8
Female	1048	62.5	59.9	65.1	45	42.5	32.2	53.4
**Age group (years)**								
18–21	445	10.3	9.1	11.6	46	42.5	31.3	54.5
22–24	234	5.8	4.8	7.0	30	26.1	17.8	36.6
25+	976	83.9	82.1	85.5	22	31.4	20.5	44.8
**Race/ethnicity**								
White, Non-Hispanic	785	55.9	52.1	59.6	46	48.2	38.1	58.4
Other	870	44.1	40.4	47.9	52	51.9	41.6	61.9
**Education**								
Less than high school or GED	290	16.2	14.1	18.5	10	8.95	5.1	15.2
High school diploma	433	25.4	21.9	29.3	20	15.5	9.5	24.4
Some college or higher	932	58.4	54.4	62.2	68	75.6	65.5	83.4
**Current product use**								
Every day	-	-	-	-	7	6.9 ^	3.5	13.3
Some days	-	-	-	-	91	93.1	86.7	96.5
**Past 30-day use of marijuana**								
No	1632	99.4	99.0	99.7	72	73.7	62.9	82.2
Yes	20	0.6	0.3	1.0	25	26.3	17.8	37.1
	***N***	**Mean**	**95% confidence interval**		***N***	**Mean**	**95% confidence interval**	
**SHS Exposure** (hours, past 7 days)	1655	1.9	1.4	2.4	98	4.8	2.3	7.2
**Creatinine level**	1646	128.7	123.0	134.4	98	159.1	141.5	176.6

Percentages are weighted; frequencies are not weighted. Confidence intervals are calculated using the delta method. * Statistically significant differences were detected in examining tobacco use status by sex, age, education, and past 30-day MJ use (Pearson *X²* test, *p* < 0.05). SHS exposure and creatinine level significantly varied based on tobacco user status (one-way ANOVA, *p* < 0.05). ^ Estimate should be interpreted with caution because it has low precision. It is based on a denominator sample size of less than 50, or the coefficient of variation of the estimate is larger than 30%. Data excluded if creatinine level ≤ 10 or ≥ 370 mg/dL.

**Table 3:** Geometric mean urinary biomarker concentrations and 95% CI (creatinine adjusted) by hookah use status.

Biomarkers of Exposure	Never Users of Tobacco Products (*n* = 1655)		% Above LOD	Exclusive, Established, Past 30-day Hookah User (*n* = 98)	% Above LOD
Cotinine (COTT) (µg/g)	*N*	1644		98	
		0.42 (0.36, 0.48)	98.8%	5.45 ^ (2.81, 10.58)	100.0%
	*N*	1633		97	
Total Nicotine Equivalents (TNE-2) (µmol/g)		0.006 (0.005, 0.007)	-	0.09 ^ (0.04, 0.17)	-
	*N*	1653		98	
4-methylnitrosamino)-4-(3-pyridyl)-1-butanol (NNAL) (ng/g)		0.92 ^ (0.82, 1.04)	50.7%	2.21 (1.59, 3.08)	83.5%
	*N*	1655		98	
3-Hydroxyfluorene (3-FLU) (ng/g)		63.98 (60.32, 67.86)	99.2%	74.22 (60.65, 90.82)	100.0%
	*N*	1655		98	
1-Hydroxypyrene (1-PYR) (ng/g)		128.14 (120.67, 136.07)	85.2%	148.30 (126.43, 173.94)	92.3%
	*N*	1652		98	
Cadmium (UCD) (ng/g)		148.77 (139.59, 158.55)	93.4%	70.23 (60.29, 81.80)	82.2%
	*N*	1653		98	
Lead (UPB) (ng/g)		351.14 (330.28, 373.31)	99.9%	271.85 (226.73, 325.96)	100.0%
	*N*	1653		98	
N-Acetyl-S-(2-cyanoethyl)-L-cysteine (CYMA) (µg/g)		1.27 (1.19, 1.36)	84.9%	2.80 (1.98, 3.98)	96.0%
*N*	*N*	1653		97	
N-Acetyl-S-(3-hydroxypropyl)-Lcysteine (HPMA) (µg/g)		262.06 (247.45,277.54)	99.6%	257.82 (216.17, 307.49)	100.0%

Frequencies are not weighted; weighted geometric mean and 95% confidence intervals (in parentheses) calculated by exp (mean of log transformed (biomarker value/creatinine value)). Total Nicotine Equivalents (TNE-2) calculated by taking molar of trans-3′-hydroxycotinine and cotinine divided by urinary creatinine. ^ Estimate should be interpreted with caution because of low reliability. It is based on a sample size of less than 50, or the coefficient of variation is greater than 30%, or the proportion of results below the limit of detection (LOD) is greater than 40%.

**Table 4:** Adjusted geometric mean ratios for urinary biomarkers of exposure by hookah use status vs. no tobacco use.

Biomarker	Exclusive, Established, Past 30-Day Hookah User (*n* = 98)			Never Users of Tobacco Products (*n* = 1655) (Ref)
		95% CI		
	Geometric Mean Ratio	Lower	Upper	
Total Nicotine Equivalents (TNE-2)	11.63 ^	5.49	24.68	1.00
Cotinine (COTT)	10.57 ^	4.94	22.61	1.00
4-methylnitrosamino)-4-(3-pyridyl)-1-butanol (NNAL)	2.29	1.52	3.46	1.00
3-Hydroxyfluorene (3-FLU)	1.28	1.01	1.63	1.00
1-Hydroxypyrene (1-PYR)	1.33	1.09	1.63	1.00
Cadmium (UCD)	0.93	0.76	1.14	1.00
Lead (UPB)	1.19	0.99	1.43	1.00
N-Acetyl-S-(2-cyanoethyl)-L-cysteine (CYMA)	1.81	1.17	2.81	1.00
N-Acetyl-S-(3-hydroxypropyl)-L-cysteine (HPMA)	1.09	0.89	1.33	1.00

Analyses are weighted and models are adjusted for transformed creatinine level (g/mL), age, sex, race/ethnicity, education attainment, past 30-day marijuana use and level of SHS exposure. ^ Estimate should be interpreted with caution because of low reliability. It is based on a sample size of less than 50, or the coefficient of variation is greater than 30%, or the proportion of results below the limit of detection (LOD) is greater than 40%.

## Supplementary Materials

The following are available online at https://www.mdpi.com/1660-4601/17/17/6403/s1. Table S1: Weighted geometric mean urinary biomarker concentrations (creatinine adjusted) by tobacco use status. Table S2: Linear regression results for biomarkers of exposure by tobacco use status.
