# Supplementary material for: Biomarkers of Exposure among USA Adult Hookah Users: Results from Wave 1 of the Population Assessment of Tobacco and Health (PATH) Study (2013–2014)
Source: Int J Environ Res Public Health. 2020 Sep 2;17(17):6403. doi: 10.3390/ijerph17176403 (PMC7504577; doi:10.3390/ijerph17176403)
Supplement: Supplementary file 1 [file ijerph-17-06403-s001.pdf]

Table S1. Weighted Geometric mean urinary biomarker concentrations (creatinine adjusted) by tobacco use status

| Biomarkers of Exposure | | Never users of tobacco products (*n* = 1,655) | % Above LOD | Exclusive, established, past 30 day hookah user (*n* = 98) | % Above LOD |
|---|---|---|---|---|---|
| **Urinary Nicotine Metabolites** | | | | | |
| Cotinine (COTT) (ug/g) | *N* | 1644 | | 98 | |
| | | 0.42 (0.36, 0.48) | 99% | 5.45^ (2.81, 10.58) | 100% |
| Total Nicotine Equivalents (2) (nmol/g) | *N* | 1633 | | 97 | |
| | | 6.29 (5.42, 7.30) | - | 86.50^ (44.44, 168.37) | – |
| Cotinine N-oxide (COXT) (ng/g) | *N* | 88 | | 33 | |
| | | 13869.70^ (6861.91, 28034.27) | 99% | 19394.30 (11679.58, 32204.58) | 97% |
| trans-3'-hydroxycotinine (HCTT) (ng/g) | *N* | 1641 | | 97 | |
| | | 689.87 (593.67,801.65) | 98% | 9180.74^ (4572.89,18431.67) | 99% |
| Norcotinine (NCTT) (ng/g) | *N* | 88 | | 33 | |
| | | 4269.73 ^ (2272.44,8021.89) | 95% | 5613.07 (3516.60,8959.37) | 97% |
| Nicotine (NICT) (ng/g) | *N* | 88 | | 33 | |
| | | 31186.49 ^ (12303.26,79052.00) | 64% | 26121.71 ^ (13758.13,49595.66) | 66% |
| Nornicotine (NNCT)^ (ng/g) | *N* | 88 | | 33 | |
| | | 3391^ (1846.85,6226.22) | 35% | 3118.67 (2068.60,4701.80) | 55% |
| Nicotine N-oxide (NOXT) (ng/g) | *N* | 88 | | 33 | |
| | | 11489.28 ^ (4238.72,31142.33) | 76% | 6949.05 ^ (3625.81,13318.22) | 66% |
| **Minor Tobacco Alkaloids** | | | | | |
| Anabasine (ANBT)^(ng/g) | *N* | 88 | | 33 | |
| | | 604.72 (347.15, 1053.40) | 29% | 306.75 (226.76, 414.96) | 32% |
| Anatabine (ANTT)^(ng/g) | *N* | 88 | | 33 | |
| | | 615.08^ (306.53, 1234.21) | 37% | 265.16 (187.68, 374.63) | 32% |
| **Arsenic and Arsenic Compounds (ug/L)** | | | | | |
| Arsenous Acid (UAS3) (ng/g) | *N* | 1653 | | 98 | |
| | | 0.33 (0.31,0.36) | 73% | 0.41 (0.34,0.5) | 91% |
| Arsenic Acid (UAS5)^ (ng/g) | *N* | 1653 | | 98 | |
| | | 0.53 (0.51,0.57) | 3% | 0.43 (0.38,0.48) | 8% |
| Dimethylarsinic acid (UDMA) (ng/g) | *N* | 1653 | | 98 | |
| | | 3.69 (3.48,3.92) | 81% | 3.6 (3.09,4.2) | 90% |
| Monomethylarsonic acid (ng/g) | *N* | 1653 | | 98 | |
| | | 0.48 (0.45,0.52) | 78% | 0.46 (0.38,0.54) | 87% |
| Total Inorganic Arsenic (ug/g) | *N* | 1653 | - | 98 | |
| | | 5360.23 (5078.07,5658.04) | - | 5221.98 (4532.44,6016.41) | - |
| **Tobacco Specific Nitrosamines (TSNAs)** | | | | | |
| 4-methylnitrosamino)-4-(3-pyridyl)-1-butanol (NNAL) (ng/g) | *N* | 1653 | | 98 | |
| | | 0.92 (0.82,1.04) | 51% | 2.21 (1.59,3.08) | 83% |
| N’-nitrosoanatabine (NAT) ^ (ng/g) | *N* | 1648 | | 98 | |
| | | 2.92 (2.74,3.11) | 1% | 2.61 (2.19,3.11) | 13% |
| N’-nitrosoanabasine (NAB) ^ (ng/g) | *N* | 1655 | | 98 | |
| | | 1.07 (1,1.14) | 2% | 0.91 (0.78,1.07) | 11% |

| | | | | | |
|---|---|---|---|---|---|
| N'-nitrosonornicotine (NNN)^ (ng/g) | *N* | 1647 | | 98 | |
| | | 1.92 (1.81,2.04) | 1% | 1.58 (1.36,1.85) | 6% |
| **Metals (ng/L)** | | | | | |
| Beryllium (UBE) ^ (ng/g) | *N* | 1653 | | 98 | |
| | | 10.53 (9.94,11.15) | 3% | 8.17 (7.22,9.23) | 6% |
| Cadmium (UCD) (ng/g) | *N* | 1652 | | 98 | |
| | | 148.77 (139.59,158.55) | 93% | 70.23 (60.29,81.8) | 82% |
| Cobalt (UCO) (ng/g) | *N* | 1653 | | 98 | |
| | | 563.81 (537.49,591.41) | 100% | 488.91 (431.58,553.85) | 100% |
| Manganese (UMN) (ng/g) | *N* | 1652 | | 98 | |
| | | 130.73 (123.58,138.29) | 47% | 94.52 (82.36,108.47) | 45% |
| Lead (UPB) (ng/g) | *N* | 1653 | | 98 | |
| | | 351.14 (330.28,373.31) | 100% | 271.85 (226.73,325.96) | 100% |
| Strontium (USR) (ug/L) | *N* | 1653 | | 98 | |
| | | 112.71 (106.77,118.97) | 100% | 109.14 (95.15,125.17) | 100% |
| Thallium (UTL) (ng/g) | *N* | 1652 | | 98 | |
| | | 171.75 (163.62,180.28) | 100% | 159.95 (143,178.9) | 100% |
| Uranium (UUR) (ng/g) | *N* | 1653 | | 98 | |
| | | 5.36 (4.85,5.93) | 85% | 4.93 (4.08,5.95) | 92% |
| **Polycyclic Aromatic Hydrocarbons (ng/mL)** | | | | | |
| 1-Naphthol or 1-hydroxynaphthalene (1-NAP) (ng/g) | *N* | 1649 | | 98 | |
| | | 1.4 (1.28,1.53) | 99% | 1.13 (0.92,1.4) | 100% |
| 2-Naphthol or 2-hydroxynaphthalene (2-NAP) (ng/g) | *N* | 1652 | | 98 | |
| | | 4.63 (4.34,4.94) | 100% | 5.07 (4.25,6.05) | 100% |
| 3-Hydroxyfluorene (3-FLU) (ng/g) | *N* | 1655 | | 98 | |
| | | 63.98 (60.32,67.86) | 99% | 74.22 (60.65,90.82) | 100% |
| 2-Hydroxyfluorene (2-FLU) (ng/g) | *N* | 1655 | | 98 | |
| | | 167.22 (158.06,176.9) | 100% | 183.01 (153.91,217.62) | 100% |
| 1-Hydroxyphenanthrene (1-PHE) (ng/g) | *N* | 1655 | | 98 | |
| | | 106.27 (101.09,111.72) | 100% | 93.86 (80.95,108.84) | 100% |
| 1-Hydroxypyrene (1-PYR) (ng/g) | *N* | 1655 | | 98 | |
| | | 128.14 (120.67,136.07) | 85% | 148.3 (126.43,173.94) | 92% |
| 2- and 3-Hydroxyphenanthrene (2-3PHE) (ng/g) | *N* | 1655 | | 98 | |
| | | 128.96 (123.03,135.18) | 100% | 124.1 (106.77,144.24) | 100% |
| **Volatile Organic Compounds** | | | | | |
| 2-Methylhippuric acid (2MHA)(ng/g) | *N* | 1589 | | 96 | |
| | | 22251.79 (20614.48,24019.14) | 92% | 17164.55 (13641.45,21597.53) | 87% |
| 3, 4-Methylhippuric acid (34MH)(ng/g) | *N* | 1651 | | 98 | |
| | | 152323 (143737.5,161421.3) | 100% | 130491.1 (106198.0,160341.4) | 100% |
| N-Acetyl-S-(2-carbamoylethyl)-Lcysteine (AAMA)(ng/g) | *N* | 1645 | | 95 | |
| | | 45308.74 (43132.9,47594.3) | 99% | 59693.49 (50328.3,70801.3) | 100% |

| | | | | | |
|---|---|---|---|---|---|
| N-Acetyl-S-(N-methylcarbamoyl)-Lcysteine (AMCA)(ng/g) | *N* | 1653 | | 97 | |
| | | 104264 (98332.0,110553.9) | 100% | 87601.85 (75633.4,101464.2) | 100% |
| N-Acetyl-S-(benzyl)-L-cysteine (BMA)(ng/g) | *N* | 1653 | | 97 | |
| | | 6124.5 (5781.5,6487.8) | 100% | 5509.2 (4661.9,6510.4) | 100% |
| N-Acetyl-S-(2-carboxyethyl)-Lcysteine (CEMA)(ng/g) | *N* | 1599 | | 90 | |
| | | 93970.34 (89669.2,98477.8) | 99% | 93779.5 (80648.35,109048.7) | 100% |
| N-Acetyl-S-(1-cyano-2-hydroxyethyl)-L-cysteine (CYHA)^(ng/g) | *N* | 1653 | | 98 | |
| | | 1817.7 (1710.1,1932.1) | 3% | 1882.9 (1498.8,2365.4) | 16% |
| N-Acetyl-S-(2-cyanoethyl)-L-cysteine (CYMA)(ng/g) | *N* | 1653 | | 98 | |
| | | 1271.5(1192.5,1355.7) | 85% | 2804.31 (1978.0,3975.8) | 96% |
| N-Acetyl-S-(3,4-dihydroxybutyl)-Lcysteine(DHBM)(ng/g) | *N* | 1481 | | 80 | |
| | | 347491.4 (335695.0,359702.2) | 89% | 309586.9 (288111.0,332663.6) | 100% |
| N-Acetyl-S-(2-carbamoyl-2-hydroxyethyl)-L-cysteine (GAMA)(ng/g) | *N* | 1581 | | 95 | |
| | | 8580.9 (8097.4,9093.3) | 38% | 9113.8 (7833.1,10603.9) | 61% |
| N-Acetyl-S-(2-hydroxyethyl)-Lcysteine (HEMA)(ng/g) | *N* | 1508 | | 80 | |
| | | 961.5 (891.3,1037.3) | 53% | 876.12 (712.38,1077.5) | 72% |
| N-Acetyl-S-(2-hydroxypropyl)-Lcysteine(HPM2)(ng/g) | *N* | 1605 | | 98 | |
| | | 32201.7 (29217.3,35490.6) | 95% | 31126.08 (24362.1,39768.1) | 99% |
| N-Acetyl-S-(3-hydroxypropyl)-Lcysteine(HPMA)(ng/g) | *N* | 1653 | | 97 | |
| | | 262065.0 (247449.6,277543.6) | 100% | 257819.9 (216169.8,307494.7) | 100% |
| N-Acetyl-S-(3-hydroxypropyl-1-methyl)-L-cysteine (HPMM)(ng/g) | *N* | 1653 | | 98 | |
| | | 440941.7 (416424.2,466899.6) | 100% | 377453.6 (333472.3,427228.0) | 100% |
| N-Acetyl-S-(4-hydroxy-2-methyl-2-buten-1-yl)-L-cysteine (IPM3)(ng/g) | *N* | 1637 | | 97 | |
| | | 3222.5 (3015.5,3443.6) | 89% | 2421.7 (2004.9,2925.1) | 91% |
| Mandelic acid (MADA)(ng/g) | *N* | 1464 | | 89 | |
| | | 127971.2 (122327.3,133875.4) | 89% | 113739.1 (102282.7,126478.6) | 99% |
| N-Acetyl-S-(4-hydroxy-2-buten-1-yl)-L-cysteine (MHB3)(ng/g) | *N* | 1653 | | 98 | |
| | | 4438.3 (4241.1,4644.6) | 99% | 3532.6(3047.5,4094.9) | 100% |
| Phenylglyoxylic acid (PGHA) | *N* | 1550 | | 94 | |

| | | | | | |
|---|---|---|---|---|---|
| | | 202278.7 (194312.3,210571.8) | 94% | 175571.8 (153871.0,200333.2) | 100% |
| N-Acetyl-S-(phenyl)-L-cysteine (PMA)(ng/g) | *N* | 1653 | | 98 | |
| | | 1006.7 (935.6,1083.3) | 68% | 810.5 (656.9,1000.1) | 72% |
| 2-Thioxothiazolidine-4-carboxylic acid (TTCA)(ng/g) | *N* | 1369 | | 87 | |
| | | 20662.5 (18334.9,23285.6) | 69% | 15518.6 (11085.9,21723.7) | 66% |

Weighted estimates; geometric mean calculated by exp (mean of log transformed [biomarker value/creatinine value]).Total Nicotine Equivalents (TNE2) calculated by taking molar sum of trans-3'-hydroxycotinine and cotinine divided by urinary creatinine. ^Estimate should be interpreted with caution because of low reliability. It is based on a sample size of less than 50, or the coefficient of variation is greater than 30%, or the proportion of results below the limit of detection (LOD) is greater than 40%.

Table S2. Linear regression results for biomarkers of exposure by tobacco use status

| **Biomarker** | **Exclusive, established, past 30 day hookah user (*n* = 98)** | | | Never users of tobacco products (*n* = 1655) (Ref) |
|---|---|---|---|---|
| | **exp** (Coef) | 95% confidence interval lower | upper | |
| **Urinary Nicotine Metabolites** | | | | |
| Urinary cotinine (COTT) | 10.57 ^ | 4.94 | 22.61 | 1.00 |
| Total Nicotine Equivalents (2) | 11.64 ^ | 5.49 | 24.68 | 1.00 |
| Cotinine N-oxide (COXT) | 1.09 ^ | 0.41 | 2.86 | 1.00 |
| trans-3'-hydroxycotinine (HCTT) | 11.69 ^ | 5.35 | 25.51 | 1.00 |
| Norcotinine (NCTT) | 1.05 ^ | 0.42 | 2.64 | 1.00 |
| Nicotine (NICT) | 0.46 ^ | 0.10 | 2.03 | 1.00 |
| Nornicotine (NNCT) | 0.71 ^ | 0.27 | 1.84 | 1.00 |
| Nicotine N-oxide (NOXT) | 0.3 ^ | 0.07 | 1.19 | 1.00 |
| **Minor Tobacco Alkaloids** | | | | |
| Anabasine (ANBT) | 0.42 ^ | 0.19 | 0.91 | 1.00 |
| Anatabine (ANTT) | 0.3 ^ | 0.11 | 0.81 | 1.00 |
| **Arsenic and Arsenic Compounds (ug/L)** | | | | |
| Arsenous Acid (UAS3) | 1.16 | 0.91 | 1.49 | 1.00 |
| Arsenic Acid (UAS5) | 1.02 | 0.98 | 1.06 | 1.00 |
| Dimethylarsinic acid (UDMA) | 1.17 | 0.99 | 1.39 | 1.00 |
| Monomethylarsonic acid | 0.99 | 0.80 | 1.23 | 1.00 |
| Total Inorganic Arsenic | 1.09 | 0.92 | 1.28 | 1.00 |
| **Tobacco Specific Nitrosamines (TSNAs)** | | | | |
| 4-methylnitrosamino)-4-(3-pyridyl)-1-butanol (NNAL) | 2.29 | 1.52 | 3.46 | 1.00 |
| N′-nitrosoanatabine (NAT) | 1.11 | 0.95 | 1.29 | 1.00 |
| N′-nitrosoanabasine (NAB) | 1.09 | 0.98 | 1.21 | 1.00 |
| N'-nitrosonornicotine (NNN) | 1.06 | 0.97 | 1.16 | 1.00 |
| **Metals (ng/L)** | | | | |
| Beryllium (UBE) | 1.01 | 0.97 | 1.04 | 1.00 |
| Cadmium (UCD) | 0.93 | 0.76 | 1.14 | 1.00 |
| Cobalt (UCO) | 0.98 | 0.88 | 1.09 | 1.00 |

| | | | | |
|---|---|---|---|---|
| Manganese (UMN) | 0.97 | 0.89 | 1.07 | 1.00 |
| Lead (UPB) | 1.19 | 0.99 | 1.43 | 1.00 |
| Strontium (USR) (ug/L) | 1.00 | 0.84 | 1.18 | 1.00 |
| Thallium (UTL) | 1.08 | 0.96 | 1.21 | 1.00 |
| Uranium (UUR) | 1.02 | 0.78 | 1.33 | 1.00 |
| **Polycyclic Aromatic Hydrocarbons (ng/mL)** | | | | |
| 1-Naphthol or 1-hydroxynaphthalene (1-NAP) | 1.00 | 0.60 | 1.64 | 1.00 |
| 2-Naphthol or 2-hydroxynaphthalene (2-NAP) | 1.19 | 0.95 | 1.50 | 1.00 |
| 3-Hydroxyfluorene (3-FLU) | 1.28 | 1.01 | 1.63 | 1.00 |
| 2-Hydroxyfluorene (2-FLU) | 1.22 | 0.99 | 1.49 | 1.00 |
| 1-Hydroxyphenanthrene (1-PHE) | 1.11 | 0.91 | 1.35 | 1.00 |
| 1-Hydroxypyrene (1-PYR) | 1.33 | 1.09 | 1.63 | 1.00 |
| 2- and 3-Hydroxyphenanthrene (2–3PHE) | 1.15 | 0.95 | 1.38 | 1.00 |
| **VOC's** | | | | |
| 2-Methylhippuric acid (2MHA) | 0.97 | 0.73 | 1.30 | 1.00 |
| 3,4-Methylhippuric acid (34MH) | 1.10 | 0.86 | 1.40 | 1.00 |
| N-Acetyl-S-(2-carbamoylethyl)-Lcysteine (AAMA) | 1.39 | 1.15 | 1.68 | 1.00 |
| N-Acetyl-S-(N-methylcarbamoyl)-Lcysteine (AMCA) | 1.25 | 1.04 | 1.50 | 1.00 |
| N-Acetyl-S-(benzyl)-L-cysteine (BMA) | 1.23 | 1.01 | 1.51 | 1.00 |
| N-Acetyl-S-(2-carboxyethyl)-Lcysteine(CEMA) | 1.22 | 1.05 | 1.42 | 1.00 |
| N-Acetyl-S-(1-cyano-2-hydroxyethyl)-L-cysteine(CYHA) | 1.21 | 0.95 | 1.55 | 1.00 |
| N-Acetyl-S-(2-cyanoethyl)-L-cysteine(CYMA) | 1.81 | 1.17 | 2.81 | 1.00 |
| N-Acetyl-S-(3,4-dihydroxybutyl)-Lcysteine(DHBM) | 1.08 | 0.98 | 1.20 | 1.00 |
| N-Acetyl-S-(2-carbamoyl-2-hydroxyethyl)-L-cysteine(GAMA) | 1.26 | 1.06 | 1.51 | 1.00 |
| N-Acetyl-S-(2-hydroxyethyl)-Lcysteine(HEMA) | 1.05 | 0.84 | 1.30 | 1.00 |
| N-Acetyl-S-(2-hydroxypropyl)-Lcysteine(HPM2) | 1.12 | 0.84 | 1.49 | 1.00 |
| N-Acetyl-S-(3-hydroxypropyl)-Lcysteine(HPMA) | 1.09 | 0.89 | 1.33 | 1.00 |
| N-Acetyl-S-(3-hydroxypropyl-1-methyl)-L-cysteine(HPMM) | 1.08 | 0.95 | 1.23 | 1.00 |
| N-Acetyl-S-(4-hydroxy-2-methyl-2-buten-1-yl)-L-cysteine(IPM3) | 0.91 | 0.71 | 1.15 | 1.00 |
| Mandelic acid (MA) | 1.00 | 0.86 | 1.17 | 1.00 |
| N-Acetyl-S-(4-hydroxy-2-buten-1-yl)-L-cysteine(MHB3) | 0.96 | 0.81 | 1.13 | 1.00 |
| Phenylglyoxylic acid (PGHA) | 1.09 | 0.95 | 1.26 | 1.00 |
| N-Acetyl-S-(phenyl)-L-cysteine (PMA) | 1.03 | 0.84 | 1.26 | 1.00 |
| 2-Thioxothiazolidine-4-carboxylic acid(TTCA) | 0.92 | 0.62 | 1.35 | 1.00 |

Analyses are weighted and models adjusted for transformed creatinine level (g/mL), age, sex, race/ethnicity, education attainment, past 30 day marijuana use and level of SHS exposure.^Estimate should be interpreted with caution because of low reliability. It is based on a sample size of less than 50, or the coefficient of variation is greater than 30%.
